# Antineoplastic Effect of Decoy Oligonucleotide Derived from MGMT Enhancer

**DOI:** 10.1371/journal.pone.0113854

**Published:** 2014-12-02

**Authors:** Tamar Canello, Haim Ovadia, Miri Refael, Daniel Zrihan, Tali Siegal, Iris Lavon

**Affiliations:** Leslie and Michael Gaffin Center for Neuro-Oncology and Department of Neurology, The Agnes Ginges Center for Human Neurogenetics, Hadassah Hebrew University Medical Center, Jerusalem, Israel; Kyung Hee University, Republic of Korea

## Abstract

Silencing of O(6)-methylguanine-DNA-methyltransferase (MGMT) in tumors, mainly through promoter methylation, correlates with a better therapeutic response and with increased survival. Therefore, it is conceivable to consider MGMT as a potential therapeutic target for the treatment of cancers. Our previous results demonstrated the pivotal role of NF-kappaB in MGMT expression, mediated mainly through p65/NF-kappaB homodimers. Here we show that the non-canonical NF-KappaB motif (MGMT-kappaB1) within MGMT enhancer is probably the major inducer of MGMT expression following NF-kappaB activation. Thus, in an attempt to attenuate the transcription activity of MGMT in tumors we designed locked nucleic acids (LNA) modified decoy oligonucleotides corresponding to the specific sequence of MGMT-kappaB1 (MGMT-kB1-LODN). Following confirmation of the ability of MGMT-kB1-LODN to interfere with the binding of p65/NF-kappaB to the NF-KappaB motif within MGMT enhancer, the efficacy of the decoy was studied *in-vitro* and *in-vivo*. The results of these experiments show that the decoy MGMT-kB1-LODN have a substantial antineoplastic effect when used either in combination with temozolomide or as monotherapy. Our results suggest that MGMT-kB1-LODN may provide a novel strategy for cancer therapy.

## Introduction

Alkylating agents such as temozolomide (TMZ) are standard first-line chemotherapy recommended for treatment of high-grade gliomas. These drugs are also used in advanced malignant melanoma and other solid neoplasms [1]–[3]. Currently the treatment of malignant tumors by alkylating agents suffers from arrested progress due to cancer cell resistance to chemotherapy. Part of this resistance is related to the presence of *O* ^6^-methyguanine-DNA-methyltransferase (MGMT), a DNA repair enzyme, that removes *O* ^6^-methylguanine adducts from the most frequent site of DNA alkylation by chemotherapeutic agents. Therefore, it is not surprising that numerous studies have demonstrated an inverse relationship between MGMT levels and survival of patients treated with alkylating drugs [1], [4]–[12]. For example, in patients with glioblastoma who are treated with TMZ the inactivation of the MGMT gene via promoter methylation is associated with improved outcome [13]. At the same time methylation of the MGMT promoter has been found to be an independent prognostic factor by itself [14]–[17]. These studies indicate that an imposed attenuation of tumor MGMT levels might prove beneficial for cancer treatment.

While aiming to enhance the efficacy of alkylating agents by reducing MGMT activity, clinical studies evaluated two pseudosubstrates: O^6^-benzylguanine (O^6^-BG) and O^6^-(4-bromothenyl)-guanine (Lomeguatrib, Patrin, KuDOS Pharmaceuticals, Ltd., Cambridge, UK). The first agent, O^6^-BG, was administered in combination with diverse alkylating drugs to treat tumors such as gliomas, melanomas, sarcomas, colon cancer and lymphomas [18]–[25]. Phase I and II clinical trials proved that the combined treatment induced substantial hematologic toxicity warranting dose reduction of the alkylating drugs. Yet, the increased toxicity was not associated with improved efficacy [22], [23], [26], [27]. Similar findings were reported for the other MGMT pseudosubstrate, lomeguatrib, ones administered in combination with TMZ for treatment of melanoma [28]–[31]. The profound dose-limiting hematologic toxicity of these two MGMT pseudosubstrates is most likely related to the total blockage of MGMT protein synthesis [32]. Thus, we hypothesized that mitigation of the transcriptional overexpression of MGMT that spares its basal transcription, might sensitize tumor cells to alkylating agents with limited affliction of the hematopoietic system.

Transcriptional control of the MGMT gene is mediated by a non-TATA box–housekeeping- gene-like promoter. The maximal activity of the promoter lies 5′ of the gene from −953 to +202 bp and consists of minimal promoter (−69 to +19), and enhancer [33]. The basal activity of MGMT is induced by the 59-bp promoter sequence located at the first exon–intron boundary of the MGMT gene. This region is sufficient to provide at least basal levels of MGMT expression both *in vitro* and *in vivo* [34]. In previous studies we have identified, two NF-kappaB binding sites within the enhancer of the MGMT gene [35]. A non-canonical NF-kappaB site at (-)763 and a canonical site at (-)93 that were designated as MGMT-kappaB1 and MGMT-kappaB2, respectively, based on their location. We have also demonstrated that p65/NF-kappaB homodimer binds specifically to both sites and is involved in MGMT gene regulation [35]. Furthermore, in our previous paper we found that either high constitutive NF-kB activity in gliomas or ectopic p65 stimulated significant cellular resistance to the alkylating agent BCNU, through induction of MGMT expression. On the other hand, inhibition of NF-kB activity by the dominant inhibitor - DNIkappaB sensitized the cells to BCNU [35].

Based on these findings, it is likely that interference with the binding of NF-kappaB to the MGMT enhancer will attenuate MGMT expression without impairing the basal expression of MGMT [33]. In the current study we evaluated the effect of such interference both *in-vitro* and *in-vivo*. In addition to the expected effect in enhancing the cytotoxicity of alkylating agent, we have also shown that such interference has independent antineoplastic effect.

## Materials and Methods

### Plasmids

For the construction of the luciferase plasmids, four copies of each kappaB site or mutant site (see below) were ligated (LigaFast rapid DNA ligation System, Promega, WI, USA) into a BglII and MluI-digested pGL2-Basic vector (Promega). The insert was generated by annealing a synthetic 5' phosphorylated oligonucleotides (Syntezza, Jerusalem, Israel). A control plasmid containing the consensus NF-kappaB sequence linked to the luciferase sequence was obtained from Clontech (pNF-kB-Luc, CA, USA). The CMVp65 and CMVdeltaNIkappaB plasmids have been previously described [35].

The sequences of the kappaB sites inserted into the luciferase vector are as follows:

MGMT-kB1-Luc: 4XGTAAAGTCCCC

MGMT-Mut-kB1-Luc: 4XGTAAAGTCGGC

MGMT-kB2-Luc: 4XGGAACACCCC

MGMT-Mut-kB2-Luc: 4XGTAAAGTCGGC

### Cell culture and transfection

The cell lines HEK293T (Human Embryo Kidney), T98G (Glioblastoma), A375P (Melanoma) and U87MG (Glioblastoma) were obtained from the American Type Culture Collection (VA, USA). The A375P and HEK293T cells were cultured in DMEM-Eagle medium supplemented with 4 mmol/L L-glutamine, 100 units/ml penicillin and 100 µg/ml streptomycin, and 10% FBS (Biological Industries, Israel). The T98G and U87MG cells were cultured in Eagle's minimum essential medium supplemented with 4 mmol/L L-glutamine, 100 units/ml penicillin and 100 µg/ml streptomycin and 10% FBS (Biological Industries). The cells were maintained in a humidified incubator at 37°C in 5% CO^2^.

The transient transfections were performed in 24 - well plates using jetPEI (Polyplus transfection, NY, USA) according to the manufacturer's instructions.

### Quantification of MGMT RNA expression by Real Time RT PCR

A375P cells were treated with increasing concentrations of MGMT-kB1–LODNs or with the control oligos. Total RNA was extracted 24 Hrs. later, and MGMT RNA expression was quantified by real-time PCR as previously described [35] using the following primers:

MGMT-F-GCAATTAGCAGCCCTGGCA; MGMT-R-CACTCTGTGGCACGGGAT.

HPRT-F-AGATGGTCAAGGTCGCAAGC; HPRT-R-ATATCCTACAACAAACTTGTCTGGAA,

PPIA-F-CGCCGAGGAAAACCGTGTAC; PPIA-R-CTCCAGTGCTCAGAGCACGA

The experiment was repeated three times in triplicate and the results are presented as the mean ± SD.

### Cell survival

To measure the viability of the cells, we used Crystal violet dye binding assay. 4% Paraformaldehyde (Gadot, Israel) was added to each well and incubated for 20 minutes at room temperature. The plates were rinsed with PBS to wash off the dead cells. 0.5% Crystal violet (Sigma-Aldrich MO, USA), was added to each well, incubated for 20 minutes, washed and dried. To solubilize the dye, 400 µl of 10% acetic acid (Gadot) was added to each well, incubated for 20 minutes. The solutions were diluted 1∶10 and read at 590 nm in a DTX 880 multimodes detectors microplate reader (Beckman Coulter, Switzerland). The average absorbance value of control was considered as 100% and the treated sample percentages were calculated by comparing the average absorbance of treated samples with the average absorbance of the control.

### Oligonucleotide (ODN) treatments

Upper strand and reverse complement LODN (30-mers), corresponding to MGMT-kB1, and the control ODNs were purchased from IDT (IA, USA). The sequences of the ODNs are as follows: MGMT-kB1-5'- TAATGGGGACTTTACGGGACTTTACAGAAT -3'

MGMT-kB1-antisense: 5'- ATTCTGTAAAGTCCCGTAAAGTCCCCATTA-3'

Control ODN sense: 5'-TAAGAGGCTAACAATGGTACAAGGTACAT -3'

Control ODN antisense: 5'- ATGTACCTTGTACCATTGTTAGCCTCTTA-3'

Each modified strand contained 6 LNA modifications inserted as in the following template (modified nucleotides are marked with “+”):

5'- +N+NNNNNNN+NNNNNNNNNN+NNNNNNNNNN+N+N -3'

### Temozolomide

#### In vitro

Temozolomide (Tocris, MN, USA) was freshly prepared by dissolving in complete cell medium. Cells were exposed to the indicated doses of TMZ 3 hrs following the transfection with the ODNs. The viability of the cells was assessed 72 hrs after TMZ treatment. Untransfected cells or cells transfected with the LODN but without TMZ treatment served as a control. All experiments were performed in triplicate with at least three biological repeats.

##### In vitro

TMZ was freshly prepared by dissolving in 10% DMSO and injected IP to athymic nude mice.

### Visualization of the LMODNs and p65 in cells

T98G and A375P were cultured in 4-well chamber slides (SPL Life Sciences, Korea) and transfected with FAM-labeled MGMT-kB1-decoy LMODNs (200 nM) (Sigma-Aldrich) using jetPEI (Polyplus transfection). After 24 hrs, the cells were fixated with 4% paraformaldehyde (Gadot) for 15 min, washed 4 times with medium and then blocked with medium containing 1% normal goat serum and 0.02% Triton X-100 (Sigma-Aldrich) for 15 minutes. The cells were then incubated for 45 min with or without rabbit polyclonal anti-NF-kB p65 (ab7970) antibody (Abcam, MA, USA), washed 3 times with medium and then incubated for 30 min with or without anti rabbit Alexa Fluor 555 anti-rabbit IgG (A31572, Life Technologies, NY, USA). Cells were then washed 3 times with medium, mounted in UltraCruz Mounting Medium containing DAPI (sc-24941 Santa Cruz, CA, USA) and visualized using a Carl Zeiss LSM5 confocal microscope.

### Luciferase assay

Cells were lysed for 15 min on ice with 200 ul luciferase lysis buffer (Promega). The luciferase assays were performed using the Promega assay kit and a luminometer (EG&G Berthold, Bad Wildbad, Germany). Luciferase activity was normalized to the beta-galactosidase activity (Promega) and calculated as the mean of triplicates from a representative experiment.

### *In vivo* A375P Melanoma tumor xenograft

#### Ethical statement

This study was carried out in accordance with the recommendations in the Guide for the Care and Use of Laboratory Animals of the National Institutes of Health. The protocol was approved by the Committee on the Ethics of Animal Experiments of the Hebrew University Medical school (Permit Number: MD-13077-5). To minimize suffering of the animals, intra tumoral injections were performed under light anesthesia (ketamine + xylazine,100 and 5 mg/kg body weight, respectively). The animals were monitored twice a week for tumor size and body weight. The animals were finally euthanized by exposure to excess of CO_2_.

Groups of 5-10 athymic nude mice were inoculated subcutaneously with 5×10^6^ Melanoma tumor cells (A375P) into the interscapular area. Tumor growth was monitored with hand-held Vernier caliper twice a week and tumor volume was estimated by calculation using the formula: (width^2^×length)/2. On day 5-6 when the tumors reached an average volume of 60-75 mm^3^, the mice were treated as indicated. In all experiments mice were sacrificed when their tumor reached a size of 900–1000 mm^3^ according to the ethical requirements of the Hebrew University Animal authorities. In the long-term *in vivo* experiment the endpoint was considered as the number of days that elapsed from tumor induction (day 0) to the day that tumor mass reached a volume of 850 mm^3^ as requested by the local animal committee in order to minimize mice suffering.

To identify the dose of TMZ that will induce a moderate tumor growth inhibition, mice were divided to 4 different groups and injected IP with TMZ as follows: a control group treated only with the vehicle (10% DMSO in PBS) and 3 groups treated with different doses of TMZ - 100 mg/Kg, 200 mg/Kg, 300 mg/Kg and 400 mg/Kg.

To evaluate the effect of the MGMT-kB1-LODN and its ability to either sensitize tumors to alkylating therapy or alternatively to serve as a monotherapy athymic nude mice were inoculated with melanoma tumor and randomized into groups of ten mice. On day 6 mice were injected IL with either 25 ug of MGMT-kB1 LODN carried by in vivo-jetPEI (Polyplus) or with vehicle (5% glucose). On day 7 IP treatment was delivered using either 100 mg/kg TMZ or vehicle (10% DMSO). On day 8 IL injection was repeated as on day 6. These experiments were repeated for three times.

In another experiment, 21 mice bearing melanoma tumors were randomized into 3 groups and were repeatedly treated every 4-5 days for 55 days with either 25 ug of MGMT-kB1 LODN or control ODN carried by in vivo-jetPEI (Polyplus) or with vehicle (5% glucose).

### Statistical analysis

Student's t-test was applied to examine the significant differences between the control and experimental data. All tests utilized were two-tailed and *P<0.05* was considered statistically significant. Survival of tumor bearing animals was measured from the date of tumor inoculation to date of end event or last follow-up. The end event was defined as the day in which tumor volume reached 850 mm^3^. Survival curves were estimated using the Kaplan-Meier product-limit methods and comparisons between treatment groups were examined by the log-rank test.

## Results

### MGMT-kB1 is the major inducer of MGMT expression following p65/NF-kappaB activation

**Figure 1:**
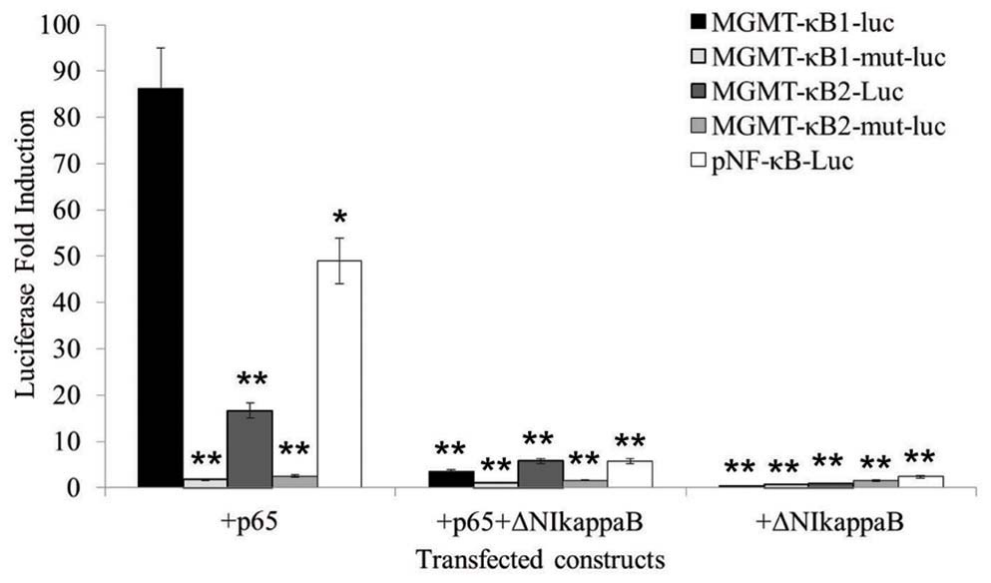
The activity induced by the NF-kappaB sites within the MGMT enhancer and their corresponding mutant sites as measured by luciferase fold induction. The HEK293T cell line was transiently transfected with a reporter gene construct either alone or with other plasmids as indicated on the graph. The CMVβ-galactosidase expression vector (CMVβgal) was included in each transfection to normalize the transfection efficiency. The observed enhancer activity is relative to the corresponding reporter plasmid transfected alone. An asterisk indicates a significant difference *(p<0.05)* compared with the control (reporter plasmid transfected alone).

To investigate whether both NF-kappaB binding motifs within the MGMT enhancer are essential to induce gene expression following NF-kappaB activation, each site was separately cloned into a luciferase reporter vector. Both vectors are comprised of either four copies of one of the two NF-kappaB binding motifs or their corresponding mutant site containing a C-to-G substitution (as described in the methods). All transfections were performed in HEK293 cells. As seen in figure 1, co-transfection of the reporter construct of the NF-kappaB1 site within the MGMT enhancer (MGMT-kB1-Luc) together with NF-kappaB/p65 generated a significant increase (86-fold) in the luciferase activity compared with the transfection of MGMT-kB1-Luc alone *(p<0.05)*. Meanwhile, the mutant site (MGMT-kB1-Mut-Luc) produced only 2-fold elevation in luciferase activity when co-transfected with kappaB/p65 (Figure 1). The reporter vector constructed from the MGMT-kappaB2 site (MGMT-kB2-Luc) increased the luciferase expression by 17-fold when co-transfected with NF-kappaB/p65, while the commercially available canonical NF-kappaB reporter construct (pNF-kB-Luc) displayed a 49-fold increase in luciferase expression. The induced activation by NF-kappaB/p65 in each of the constructs was abolished when the super repressor DeltaNI-kappaB was added to the transfection.

### MGMT-kB1 LNA modified oligonucleotides (LODN) interfere with the binding of NF-kappaB to KappaB motif within MGMT enhancer and reduce MGMT RNA levels

**Figure 2:**
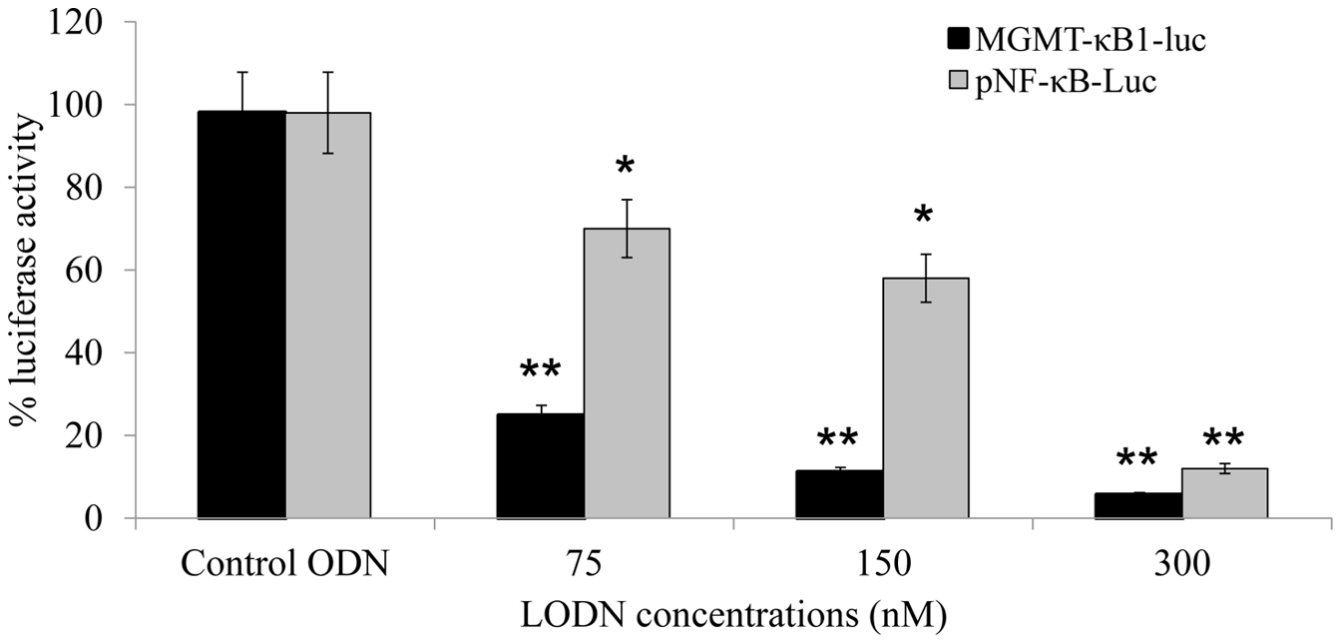
Interference with NF-kappaB binding to the MGMT-NFkB1 site using LODN (locked modified ODN). The HEK293T cell line was transiently transfected with either the kB1-MGMT-luc or pNF-kB-Luc reporter gene construct together with NFkappaB/p65. LODN corresponding to the MGMT-kB1 site were added at the indicated concentrations. The CMVβ-galactosidase expression vector (CMVβgal) was included in each transfection to normalize the transfection efficiency. The observed enhancer activity is relative to the corresponding reporter plasmid transfected with NFkappaB/p65. All concentrations of MGMT-kB1 LODN significantly reduced the levels of luciferase expression *(p<0.05)* compared with cells transfected with control ODN. The control bars indicate the average inhibition of three different concentrations of ODN as indicated for MGMT-kB1 LODN. An asterisk indicates a significant difference of *p<0.05* and a double asterisk indicates *p<0.01*.

Decoy LODN corresponding to the specific sequence of MGMT-kappaB1 were designed to study whether interference with the binding of NF-kappaB to the MGMT enhancer would attenuate P65/NFkappaB induced luciferase expression. We included LNA bases in our decoy oligonucleotide to overcome their intracellular degradation by endonucleases and exonucleases [36]. LNA modifications has been also shown to exhibit a very high thermal stability [37], unprecedented affinity and increased metabolic stability [38], [39]. HEK293T cells were co-transfected with the reporter construct MGMT-kB1-Luc and NFkappaB/p65. These cells were also co-transfected with LNA- Modified MGMT-kB1 Oligonucleotide (MGMT-kB1–LODN) at increasing doses to induce the inhibitory effect. The co-transfection resulted in a significant inhibition *(p<0.05)* of the luciferase activity in a dose-dependent manner with an inhibition of 78% and 88% induced by 75 nM and 150 nM MGMT-kB1-LODN, respectively (Figure 2). For comparison, the co-transfection of the commercially available NF-kappaB reporter construct (pNF-kB-Luc) with NFkappaB/p65 and MGMT-kB1-LODN yielded a weaker inhibition of luciferase expression (only 30% and 42% inhibition for 75 nM and 150 nM LODN, respectively), which was also statistically significant *(p<0.05).* We used a control oligonucleotide in three different concentrations as the MGMT-kB1-LODN and none of them showed a significant inhibition (Figure 2).

**Figure 3:**
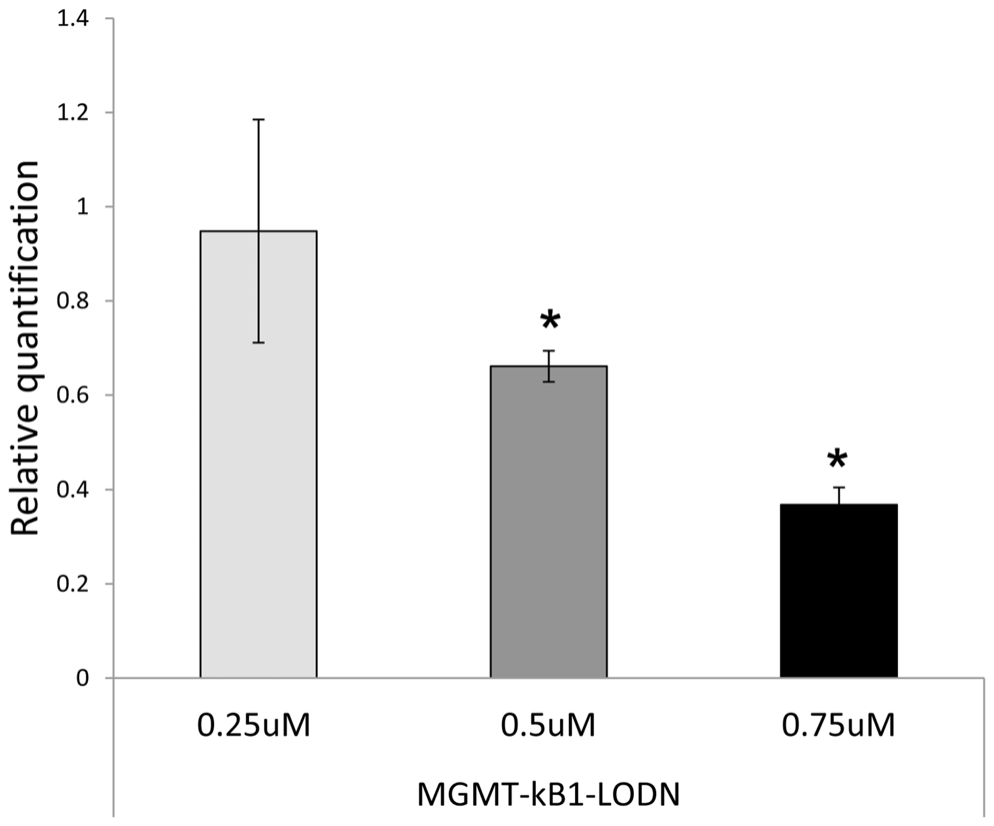
MGMT-kB1-LMODN decoy reduces the levels of MGMT RNA expression. A375P cells were transiently transfected with either MGMT-kB1-LMODN or the control oligonucleotides. At 24 hrs post transfection, the changes in MGMT expression levels were analyzed by real-time RT-PCR. The fold-change (y axis) represents the relative expression of the MGMT mRNA versus that of cells transfected with the control oligonucleotides. An asterisk indicates a significant difference *(p<0.05)* compared with the control.

To verify whether the interference in the binding of NF-kappaB to MGMT enhancer induces transcriptional inhibition of MGMT, we measured the levels of MGMT mRNA by real time RT PCR in the A375P cell line following exposure to the decoy LMODNs. MGMT mRNA expression was inhibited in a dose dependent manner in cells treated with MGMT-kB1-LODN as compared to cells treated with the same amount of the control oligonucleotides. Treatment with 500 and 750 nM induced an mRNA inhibition of 0.34 and 0.64 respectively *(P<0.05)*. (Figure 3).

### MGMT-kB1-LODN are sequestered in the cytoplasm and co-localized with endogenous NF-kappaB/P65

**Figure 4:**
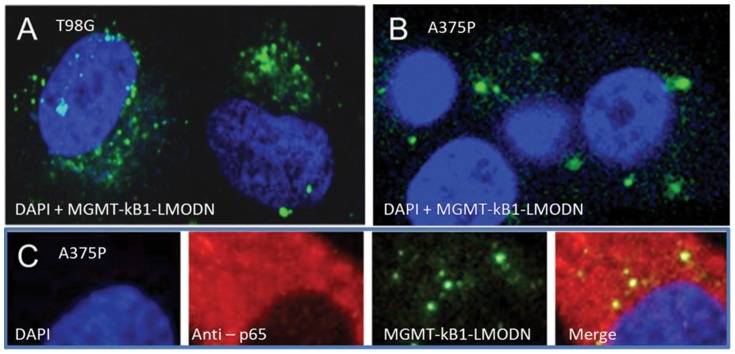
MGMT-kB1-LMODN are co-localized with NFkappaB/p65 in the cytoplasm. The cytoplasmic localization of MGMT-kB1-LMODN is demonstrated by FAM-labeled transfected into T98G cells (A) and A375P cells (B & C). Co-localization of FAM-labeled MGMT-kB1-LODN to NFkappaB/p65 is demonstrated using immunofluorescent anti-p65 antibody (C). MGMT-kB1-LMODN is visualized in green; the nuclei labeled with DAPI are visualized in blue, NFkappaB/p65 is visualized in red and co-localization of NFkappaB/p65 and MGMT-kB1-LODN is visualized in yellow.

The subcellular distribution of MGMT-kB1-LODN was studied by transfection of T98G and A375P cells with 6-carboxyfluorescein (FAM) labeled MGMT-kB1-LODN (Figure 4A, B). Visualization of the cells by confocal microscope, 24 hrs and 48 hrs post-transfection revealed that the distribution of the MGMT-kB1-LODN in T98G and A375P cells was essentially cytoplasmic. Furthermore, treating A375P MGMT-kB1-LODN transfected cells with anti P65 antibodies indicated a 90% co-localization of MGMT-kB1-LODN and p65 in the cytoplasm (Figure 4C).

### MGMT-kB1-LODN augments temozolomide-induced cell killing and has an antineoplastic effect as a monotherapy both *in-vitro* and *in-vivo*

The potential of the MGMT-kB1-LODN to enhance cell killing was studied *in vitro* using two glioma cell lines (T98G and U87) and a melanoma cell line (A375P).

**Figure 5:**
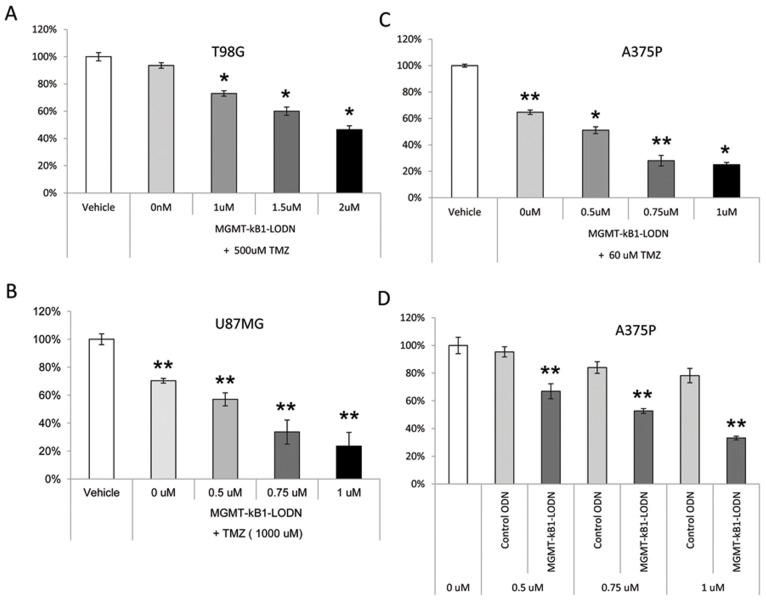
The cytotoxic efficacy of MGMT-kB1-LODN treatment given either in sequence with Temozolomide or as a monotherapy in three tumor cell lines. The effect of combination treatment with MGMT-kB1-decoy LMODNs and TMZ in three tumor cell lines (A) T98G, (B) U87MG and (C) A375P. The cells were transfected with the indicated concentrations of MGMT-kB1-LODN, and 3 hrs later were treated with the indicated doses of TMZ. The percentage of cell survival was evaluated 72 hrs later. (D) The effect of MGMT-kB1-LODN as a monotherapy. Each point represents the average viability percentage ± SEM. (A, B) An asterisk indicates a significant difference of *p<0.05* and double asterisk indicates *p<0.01*, between cells treated with MGMT-kB1-LODN and untreated cells. (D) The results are expressed as percentage of cell survival compared with cells treated with control ODN.

The three cell lines manifested an enhanced cell killing effect following exposure to temozolomide when first transfected with MGMT-kB1-LODN. TMZ was added 3 hours following the transfection with MGMT-kB1-LODN. The dose of TMZ was selected by its ability to induce a cell killing effect that did not exceed 30% when used as a single agent in each cell line. These TMZ doses varied between the three lines and were 500 uM for T98G, 60 uM for A375P and 1000 uM for U87MG cells (Figure 5 A-C). Concentrations of 0.75 uM of MGMT-kB1-LODN for U87 and A375P cell lines, and of 2 uM for T98G enhanced cell killing by approximately 2 folds. Furthermore, MGMT-kB1-LODN proved to have a cytotoxic effect when used as a monotherapy. In U87 and T98G cells concentrations of 1 uM and 2 uM of MGMT-kB1-LODN induced a significant *(p<0.05)* cell killing of 34% and 48% respectively when administered as monotherapy (data not shown). In A375P cells 0.75 uM and 1 uM of MGMT-kB1-LODN led to cell killing of 33% and 55% respectively *(p<0.01)*. This effect appears to be specific, as exposure to the control ODN had no impact on cell viability (Figure 5D).

**Figure 6:**
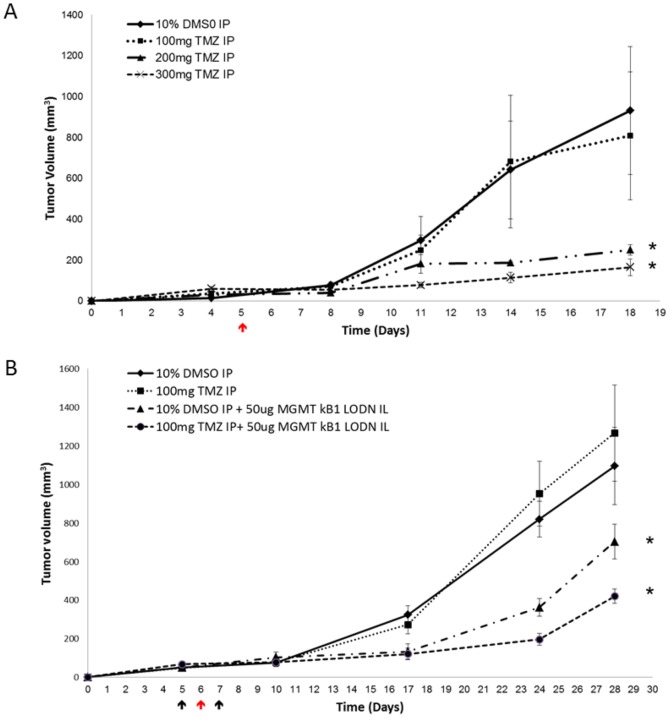
*In-vivo* efficacy of the compound drug (kB1-MGMT-LODN and the carrier) with or without temozolomide in A375P human melanoma xenografts. Athymic nude mice were inoculated subcutaneously with 5*10^6^ tumor cells (A375P) and randomized into treatment groups. (A) a single dose of IP treatment (indicated by red arrows) was administered as follows: 10% DMSO (control) or TMZ at a dose of 100 mg/kg or 200 mg/Kg or 300 mg/kg. (B) Treatment started on day 5 when the tumors grew to an approximate size of 75 mm^3^. Mice were injected IL (indicated by black arrows) with 25 ug of MGMT-kB1 LODN or with vehicle (5% glucose) on days 5 and 7. On day 6 IP treatments (red arrow) were given with either 100 mg/kg TMZ or with vehicle (10% DMSO). An asterisk indicates a significant difference *(p<0.05)* between the treated and control group (10% DMSO). Each point represents the average tumor size ± SEM.

The next step was to evaluate the *in-vivo* efficacy of MGMT-kB1-LODN and its ability to either sensitize tumors to alkylating therapy or act as a monotherapy. First, we evaluated the potency of different concentrations of TMZ on A375P xenografts. A375P Melanoma cells were injected subcutaneously into the interscapular area of athymic nude mice. On day 5 post inoculation, mice were treated with either TMZ or with vehicle (10% DMSO) by Intraperitoneal (IP) injections. Tumor volume was subsequently estimated twice a week. This experiment demonstrated that a TMZ dose of 100 mg/kg had no effect on tumor growth, while doses of 200 mg/kg and 300 mg/kg significantly restrained tumor progression in comparison to vehicle (Figure 6A).

The next series of experiments tested the effect of MGMT-kB1-LODN treatment either as a monotherapy or in combination with TMZ. Treatment started at day 6, when tumor volume reached 60 mm^3^, with intratumoral (Intralesional - IL) injection of 25 ug of MGMT-kB1-LODN. On the next day an IP dose of 100 mg/kg TMZ was administered to be followed one day later by an additional IL dose of MGMT-kB1-LODN. The results were compared to treatment with vehicle (10% DMSO). As expected, treatment with 100 mg/kg TMZ was ineffective while treatment with MGMT-kB1-LODN either as a monotherapy or in combination with TMZ significantly inhibited tumor growth *(p<0.05)* (Figure 6B).

**Figure 7:**
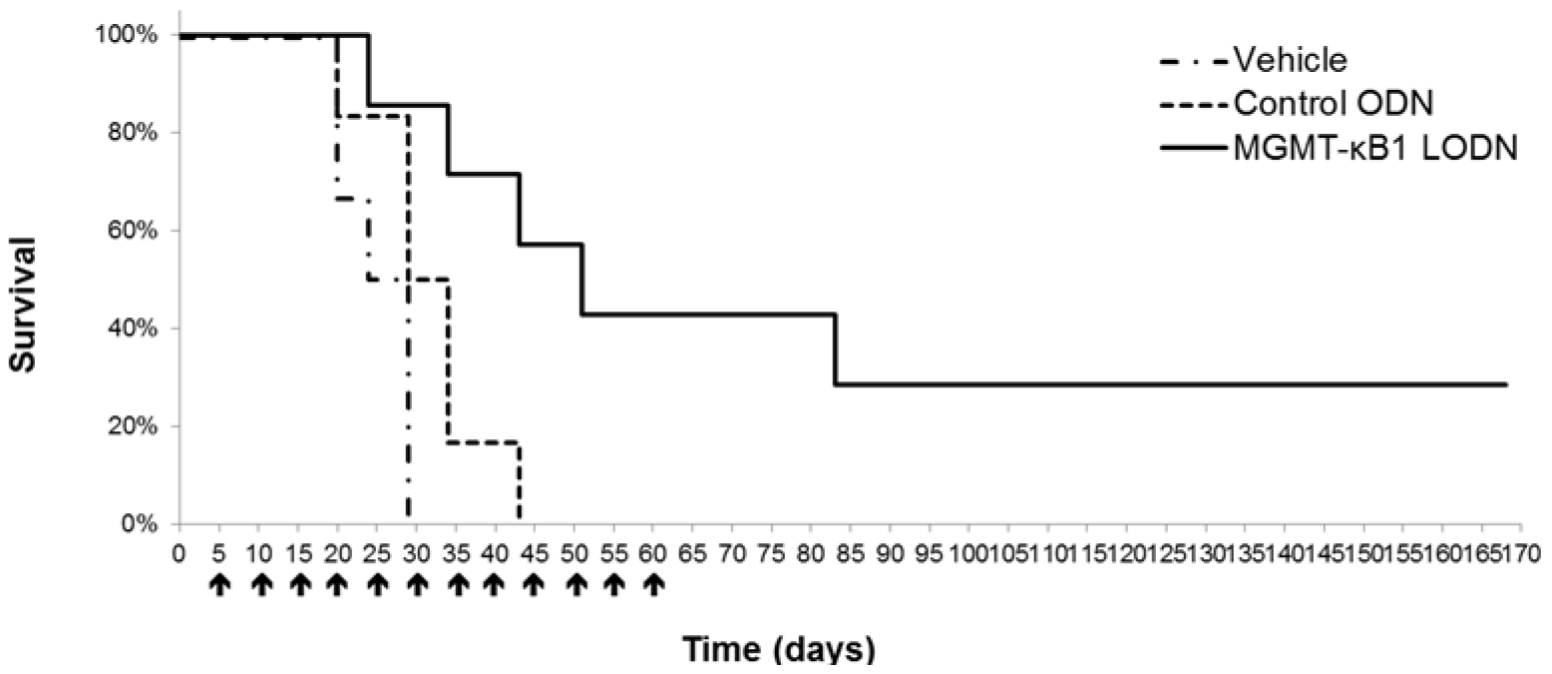
*In-vivo* efficacy of long-term repetitive intralesional administration of the compound drug (kB1-MGMT-LODN and the carrier) in A375P human melanoma xenografts. Kaplan Meier survival curve of mice bearing A375P subcutaneous xenograft. IL treatment with either 25 ug of MGMT-kB1-LODN or with control ODN or with vehicle (5% glucose) was started once tumor volume approached 75 mm^3^ (on day 6 post inoculation) and treatment was repeated every 4 to 5 days for up to 55 days after tumor induction.

Fig 5B demonstrates that the suppressive effect of MGMT-kB1-LODN treatment on tumor growth diminished about 10 days after the last injection. That led us to study the long-term effect of MGMT-kB1-LODN monotherapy using a repetitive IL dosing schedule of every 4 to 5 days. Mice bearing A375P melanoma tumor cells were treated by IL injection with either 25 ug of MGMT-kB1 LODN or control ODN or vehicle (5% glucose). IL injections were started once tumor volume approached 75 mm^3^ (on day 6 post inoculation) and were repeated every 4 to 5 days for up to 55 days after tumor induction. Figure 7 shows the survival Kaplan Meier curves of the three treatment groups. Survival time was considered as the number of days that elapsed from tumor induction (day 0) to the day that tumor mass reached a volume of 850 mm^3.^ We selected the latter as the end event because animals had to be sacrificed shortly after according to the regulation of the animal's ethic committee. A significant difference (*p<0.01)* in tumor volume was observed between the group treated with MGMT-kB1 LODN and both control groups. Furthermore, two out of the seven mice who were treated with IL MGMT-kB1 LODN demonstrated tumor regression by day 55 and no tumor recurrence was observed five months after the end of treatment.

## Discussion

This study proved that our specific MGMT-kB1-LODN intervene in NF-kappaB binding to MGMT enhancer, reduces MGMT RNA levels and induce a cytotoxic effect both *in-vitro* and *in-vivo*. The observed antineoplastic effect of this agent was independent of exposure to an antineoplastic alkylating drug such as TMZ. In addition, we showed that a sequential exposure to MGMT-kB1-LODN and TMZ can enhance the antineoplastic effect at the tested schedule. These findings go along with previous clinical observations that silencing of the enzyme MGMT, correlates with a better response to treatment and with overall survival of cancer patients [1], [4]–[14]

Our aim was to target the transcriptional activity of MGMT in tumors with decoy oligonucleotides. We designed the LNA modified decoy oligonucleotides (LODN) corresponding to the specific motif of the non-canonic NF-KappaB (MGMT-kappaB1) within MGMT enhancer based on our findings that it is the major inducer of MGMT expression following NF-kappaB activation. Also, we demonstrated that MGMT-kB1-LODN interferes in a dose-dependent manner with the binding of p65/NF-kappaB to NF-kappaB motif within MGMT enhancer and induces transcriptional inhibition of MGMT. The repressive effect of this specific MGMT-kB1-LODN decoy against a canonical NF-kappaB site (pNF-kB-Luc) was remarkably inferior when compared with the extent of inhibition it induced on the MGMT-kB1 site; this might indicate that MGMT-kB1-LODN have a higher efficiency for blocking MGMT than other NF-kappaB target genes. Furthermore, the specific inhibitory activity of MGMT-kB1-LODN was demonstrated by the lack of suppressive effect observed with the control ODNs.

The confocal microscopy results displayed that the FAM-labeled MGMT-kB1-LODN are essentially distributed in the cell cytoplasm. We have previously shown by EMZA that MGMT-kB1 site within the MGMT enhancer binds preferentially to NF-kappaB/p65 homodimers and that this homodimer plays a major role in the induction of MGMT expression [35]. Here we show that MGMT-kB1-LODN localize with p65 in the cytoplasm. In resting cells I-kappaB masks the DNA binding and nuclear localization sequences of NF-κB/p65, however, there is a dynamic equilibrium between I-kappaB -bound and unbound NF-kappaB [40]. Thus it might be assumed that MGMT-kB1-LODN binds to I-kappaB unbound p65 in either resting or activated cells. Therefore, it is likely that the effect of MGMT-kB1-LODN arise from its binding to dimers of P65 within the cytoplasm, which consequently prevents their translocation into the nucleus. This observation is similar to previous findings that detected cytoplasmic localization for other decoy oligonucleotides such as STAT3 [41] NF-kappaB [42] and AP-1 [43], which also induced their activity by hindering nuclear localization of the transcription factor.

MGMT-kB1-LODN augmented TMZ-induced cell killing and proved to have a substantial antineoplastic effect as monotherapy both *in-vitro* and *in-vivo*. Similarly, the MGMT inhibitor O^6^-BG and other DNA damage repair enzyme inhibitors, such as the poly (ADP-ribose) polymerase (PARP) inhibitors, demonstrated efficacy as monotherapy [44]–[46]. For O^6^-BG it was found that the cell killing effect is not mediated only by downregulation of MGMT but also through cell cycle regulatory proteins [47]. These observations suggest that MGMT is involved in cell cycle and DNA replication components and imply that, apart from its function as a DNA repair enzyme; MGMT may also exert other functions that are important for cancer cell survival. It should be noted that a large number of cancer cells display high constitutive activity of NF-kappaB and consequently high expression levels of MGMT [35]. In these cancer cells, the decoy LODN should induce cell killing predominantly through inhibition of NF-kappaB-induced MGMT expression, but also it might exert its effect through inhibition of other NF-kappa B-regulated genes such as anti-apoptotic (IAP1, IAP2, XIAP Bcl-2, Bcl-xL, and survivin) [48], [49] and proliferative genes (cyclin D1, cyclooxygenase-2, and c-Myc) [50]–[52]. The effect of the decoy on the other NF-kappa B target genes is probably less pronounced due to a much more repressive effect on the non-canonical NF-kappaB site within the MGMT enhancer, than on a canonical NF-kappaB reporter. Further experiments are required to determine the effect of the decoy on other NF-kappa B related and un-related genes.

The *in-vivo* efficacy of long-term repetitive administration of the compound drug (kB1-MGMT-LODN and the carrier) in A375P human melanoma xenografts is particularly interesting. This approach goes along with the concept that controlling tumor growth by treating it as a chronic disease may be meaningful [53]. The regimen we used for long-term therapy is similar to previous studies[54] showing that mice bearing subcutaneous human xenograft tumors could respond to low doses of chemotherapy, even when they displayed acquired resistance to the same agent given in a conventional way. The mechanisms of action of prolonged low-dose, minimally toxic antineoplastic therapy is complex and may lead to induction of tumor dormancy [55]. Likewise, the sustained effect observed in our study is promising and warrants further investigations of maintenance therapy regimens. Future studies should explore the prolonged MGMT-kB1-LODN therapy delivered either as a monotherapy or in combination with variety of other agents. We already showed that integration with TMZ is feasible and probably effective, yet the optimal schedule and choice of the best fitting agent for a combined therapy requires further studies.
